# Ultrasound Diagnosis of Acute Appendicitis in Pediatrics: A Community Hospital Network Experience

**DOI:** 10.7759/cureus.82854

**Published:** 2025-04-23

**Authors:** Ravi Uppal, Cornelia Muntean, Christopher Raio, Halina Borowski, Cameron Kalin, Jillian Emanuel, Lauren R Klein

**Affiliations:** 1 Emergency Medicine, Good Samaritan University Hospital, West Islip, USA

**Keywords:** diagnosis of acute appendicitis, pediatric ct, pediatric ultrasound, point-of-care-ultrasound, trans-abdominal ultrasound

## Abstract

Background

Ultrasound has emerged as the preferred initial imaging modality for diagnosing acute appendicitis in children, but most data supporting its use come from academic children’s medical centers. The purpose of this investigation is to describe ultrasound utilization for diagnosing acute appendicitis in children at a large community hospital network.

Methods

This was a multicenter retrospective observational cohort study and chart review of pediatric patients with acute appendicitis diagnosed in the emergency department (ED). The primary outcome of this study was the proportion of patients diagnosed with ultrasound as the sole imaging modality, presented as a population proportion with a 95% confidence interval.

Results

There were 450 patients included. The mean age was 10 years (range 3-17). Among the 450 patients, there were a total of 713 imaging studies. Only 213 (47.3%) patients had a single imaging test ordered during the ED encounter, and only 52 patients (11.6%) had an ultrasound during the encounter as the only imaging modality. The initial study ordered was a CT scan in 184 (40.9%), an ultrasound in 172 (38.2%), and an X-ray in 94 (20.9%). Sixty-two of 172 (36.0%) ultrasounds were positive for appendicitis. Of the 62 positive studies, 11 (17.7%) had a confirmatory CT scan.

Conclusion

In this community hospital network, patients had multiple imaging modalities ordered to confirm the diagnosis of acute appendicitis. Less than half had an ultrasound ordered as the initial imaging modality, suggesting its use is not as widespread as academic data may indicate. Despite a positive diagnostic ultrasound, some patients went on to have a confirmatory CT scan, which provides further opportunity to improve.

## Introduction

Appendicitis is the most common cause of an acute surgical abdomen in children and is, therefore, one of the most important pathologies to assess for as an emergency physician. Historically, appendicitis was diagnosed by history and physical examination alone; however, with the advent of advanced imaging, children are rarely taken to the operating room based on clinical assessment alone [1,2].

The two modalities used for diagnosing acute appendicitis are computed tomography (CT) and ultrasound. CT offers a high sensitivity and specificity for the diagnosis of appendicitis; however, it has the disadvantage of imparting ionizing radiation, which is a particular concern in the pediatric population [3]. Ultrasound uses no ionizing radiation, but its limitations are that it is highly operator dependent, with potential for non-diagnostic images and non-visualization of the appendix [4-7]. Despite the potential for a non-diagnostic study, ultrasound has arguably emerged as the preferred initial imaging modality for the diagnosis of acute appendicitis in children due to its safety profile compared to CT [8,9]. However, in actual clinical practice, it is not clear if community emergency medicine has adopted this practice.

The purpose of this study is to determine the proportion of pediatric patients with acute appendicitis who were diagnosed with ultrasound as the sole imaging modality in a large community hospital network.

## Materials and methods

Study design and setting

This is a retrospective, observational, multicenter study of pediatric patients who were diagnosed with appendicitis in the emergency department (ED) from 2018 to 2023. The study includes data from six community hospitals, part of the Catholic Health regional healthcare system. Five hospitals are staffed by emergency physicians only, and one hospital has a dedicated pediatric ED staffed by a combination of Emergency Medicine (EM) physicians and pediatric EM physicians.

Study procedures and outcomes

To identify eligible encounters, we queried the electronic medical record (EMR) for all pediatric patients (age <18) with an ED diagnosis of acute appendicitis. Patients were excluded if they were sent to the ED with an established diagnosis by outpatient imaging, were transferred from an outside facility, or had appendicitis diagnosed after admission to an inpatient unit.

Study investigators reviewed all of the charts to confirm an ED diagnosis of acute appendicitis and to confirm that the patient met all of the inclusion and exclusion criteria as outlined above. Through chart review, we identified patient age, gender, race, BMI, ED length of stay, ED disposition, surgeon training (general, pediatric), and ED provider level of training as EM, Pediatric Emergency Medicine (PEM), or Pediatrics. We collected data on all imaging tests obtained, including what modality the test was (CT scan, ultrasound, X-ray) and their corresponding results. Our EDs do not use abdominal MRI to evaluate for appendicitis; therefore, that was not included as an option. Imaging results were classified as “negative for acute appendicitis” if the imaging test was able to visualize a normal appendix and excluded appendicitis, “positive for appendicitis” if it was explicitly diagnosed acute appendicitis, “non-diagnostic for acute appendicitis” if the abdominal imaging test did not evaluate for appendicitis, “unable to visualize the appendix” if the test was unable to visualize the appendix, “equivocal for appendicitis” if the imaging result mentioned any abnormal or equivocal findings possibly related to appendicitis without definitely diagnosing appendicitis, or “positive for another important finding” if other pathology was identified on the imaging test.

We reported the total number of imaging studies obtained in the cohort. We report the rates at which tests were ordered initially (CT scan, ultrasound, or X-ray). We also report the frequencies of different modalities for the second and third imaging tests ordered. The primary outcome of this study was the proportion of patients diagnosed with ultrasound as the sole imaging modality. Secondary outcomes of interest included rates of ultrasound used as the first imaging modality, rates of ultrasound as the first imaging modality that was positive for appendicitis, and the number of positive initial ultrasound findings with subsequent CT scans. We also performed a secondary analysis of imaging modality selection based on patient age, gender, ED provider training, and surgeon specialty. 

Analysis

All data were collected using Research Electronic Data Capture (REDCap) electronic data capture tools. REDCap is a secure, web-based software platform used for standardized data collection [10,11]. Data were then analyzed utilizing Stata (StataCorp, College Station, TX) and presented descriptively, including means, medians, counts, and proportions as appropriate. We compared ordering patterns stratified by various patient variables and provider variables (age, gender, ED provider training, and surgeon specialty) and analyzed these by a chi-squared test for proportions with an alpha of 0.05, which was considered statistically significant. We included the adjusted standardized residuals and chi-squared statistic for each comparison as well.

## Results

A total of 566 patients were identified in the EMR query, of which 450 met the inclusion criteria for this study. There were 41 patients excluded because they did not have appendicitis, 15 patients had imaging done as an outpatient, 58 were transferred from another hospital, and two were diagnosed with appendicitis during admission.

In the final included cohort, the mean age was 10 years, and 172 (38.2%) were female. Additional demographics of the cohort are depicted in Table 1. A total of 713 imaging studies were ordered on the 450 patients in the cohort. Only 213 (47.3%) patients had a single imaging test ordered, and only 52 patients (11.6%) just had an ultrasound during the encounter. 

**Table 1:** Demographic and clinical data

Demographic and clinical variables	N = 450
Male	278 (61.8%)
Age (mean, range)	10 (3-17)
Ethnicity (Hispanic or Latino)	207 (46.0%)
BMI (mean, range)	19 (14-28)
ED length of stay (mean, range)	6 (1-20)
ED provider level of training	
PEM	153 (34.0%)
EM	117 (26.0%)
Pediatrics	180 (40.0%)
Surgeon specialty	
General surgery	322 (71.6%)
Pediatric surgery	128 (28.4%)
Appendectomy	436 (96.9%)
Hospital length of stay (mean, range)	2 (1-24)
Radiology ordering variables	
Initial imaging modality ordered	
CT	184 (40.9%)
Ultrasound	172 (38.2%)
X-ray	94 (20.9%)
Second imaging modality ordered (N = 213)	
CT	156 (73.3%)
Ultrasound	46 (21.5%)
X-ray	11 (5.2%)
Third imaging modality ordered (N = 52)	
CT	50 (96.2%)
Ultrasound	1 (1.9%)
X-ray	1 (1.9%)

The initial test ordered was as follows: CT (184, 40.9%), ultrasound (172, 38.2%), and X-ray (94, 20.9%). Among the 184 patients who had a CT scan ordered first, only one (0.5%) went on to have a second test (which was an ultrasound). Among the 172 patients with an ultrasound ordered first, 108 (62.8%) went on to have a CT scan. Among the 94 patients with an X-ray first, 45 (47.9%) went on to have an ultrasound, and 49 (52.1%) went on to have a CT scan. The overall positive study rate (regardless of modality) on the initial test was 227 (50.4%). 

Table 2 explores ordering patterns for the initial imaging modality choice. There were statistically significant differences in initial testing ordering patterns for age, ED provider level of training (PEM, EM, Pediatrics), and surgeon level of training (general surgery, pediatric surgery), but not for gender. 

**Table 2:** First imaging modality choice stratified by demographic variables Statistical testing by chi-squared. An alpha of 0.05 is considered significant and marked with an *. p-values are only presented for entire categories of variables; empty data cell place holders are represented by hyphens. The numbers in {curly brackets} are the adjusted standardized residuals.

	First imaging modality choice			
	CT Scan	Ultrasound	X-ray	χ^2^ and p-value
Overall	184 (40.9%)	172 (38.2%)	94 (20.9%)	-
Gender				χ^2^ = 0.7, p = 0.7
Female (N = 172)	67 (38.9%) {-0.7}	70 (40.6%) {0.9}	35 (20.3%) {-0.2}	-
Male (N = 278)	117 (42.0%) {0.7}	102 (36.7%) {-0.9}	59 (21.2%) {0.2}	-
Age				χ^2^ =24.1, p < 0.001*
<6 years old (N = 50)	9 (18.0%) {-3.5}	24 (48.0%) {1.5}	17 (34.0%) {2.4}	-
6-13 years old (N = 323)	132 (40.9%) {-0.02}	119 (36.8%) {-1.0}	72 (22.3%) {1.2}	-
14-18 years old (N = 77)	43 (55.8%) {2.9}	29 (37.7%) {-0.1}	5 (6.5%) {-3.4}	-
Initial ED provider training				χ^2^ = 124.5, p < 0.001*
Pediatric EM (N = 153)	25 (16.4%) {-7.6}	94 (61.4%) {7.3}	34 (22.2%) {0.5}	-
EM (N = 117)	94 (80.3%) {10.1}	11 (9.4%) {-7.4}	12 (10.3%) {-3.2}	-
Pediatrics (N = 180)	65 (36.1%) {-1.6}	67 (37.2%) {-0.4}	48 (26.7%) {2.4}	-
Surgeon level of training				χ^2^ = 15.5, p < 0.001
General surgeon (N = 322)	150 (46.6%) {3.9}	113 (35.1%) {-2.2}	59 (18.3%) {-2.1}	-
Pediatric surgeon (N = 128)	34 (26.6%) {-3.9}	59 (46.1%) {2.2}	35 (27.3%) {2.1}	-

Ultrasound was performed 218 times in this cohort. The ultrasound study results were as follows: positive for acute appendicitis: 69 (31.7%), negative for acute appendicitis: three (1.4%), unable to visualize appendix: 123 (56.5%), and non-diagnostic/equivocal for appendicitis: 23 (10.6%) (Table 3).

**Table 3:** Ultrasound radiology results Data on all 218 ultrasounds performed (regardless of the order of the study).

Ultrasound radiology results	N = 218
Positive for acute appendicitis	69 (31.7%)
Negative for acute appendicitis	3 (1.4%)
Unable to visualize appendix	123 (56.5%)
Non-diagnostic/equivocal for appendicitis	23 (10.6%)

Regarding our primary outcome, there were 62 patients (13.7%) who had an ultrasound done as the first testing modality and were positive for acute appendicitis. Among the 62 positive studies, 11 of these patients (17.7%) went on to have a CT scan despite the positive result. This yielded a final number of 52 total patients (11.6%) who had a positive ultrasound as the only diagnostic test ordered (95% confidence interval 8.9-14.9%) (Table 4).

**Table 4:** Primary outcome results

Primary outcome results	
Ultrasound as first imaging modality positive for appendicitis	62/450 (13.7%)
Number of positive initial ultrasounds with subsequent CT scans	11/62 (17.7%)
Total number with positive ultrasound as only diagnostic test ordered	52/450 (11.6%), 95% CI (8.9-14.9%)

## Discussion

In this community hospital network, the vast majority of patients had multiple imaging modalities obtained to confirm a diagnosis of acute appendicitis. CT was the most common initial imaging modality ordered and was sometimes ordered even after a positive ultrasound for appendicitis. This suggests an overall preference and reliance on CT imaging in this community hospital network setting, despite data in tertiary and pediatric centers shifting toward ultrasound utilization [12,13].

Other factors with regard to initial imaging tests ordered may be the training of the provider ordering the test, as well as the type of surgeons staffing the hospital. There have been studies that have shown that length of stay, risk of infectious complications, and risk of readmission do not differ regardless of whether they are operated on by pediatric surgeons or adult surgeons, suggesting resources currently consumed by transferring children to hospitals with access to pediatric surgeons could be allocated elsewhere [14]. However, those studies did not evaluate how the imaging choices were affected by the type of surgeon. In our study, most cases in which appendicitis was diagnosed with ultrasound and still went on to have a CT scan were due to request by the adult surgeons. This finding indicates that there may be potential to better educate adult surgeons who manage pediatric appendicitis so that they do not rely as heavily on CT as the sole modality, given the validity of positive ultrasounds in diagnosing appendicitis [15].

In addition, pediatric centers, which are primarily staffed by PEM providers, have much higher rates of US-diagnosed appendicitis and lower rates of CT utilization, as evidenced by studies showing that hospital type can predict the use of CT imaging for the diagnosis of appendicitis. [12,16-18]. Our study was consistent with the literature in that PEM providers were more likely to order ultrasounds as the primary study compared to EM providers. These practice differences between PEM and EM providers, which have been demonstrated in numerous studies, show us the potential for improvement with standardization and clinical pathways for the diagnosis of pediatric appendicitis, which could potentially bridge some of the gaps in imaging patterns [19-21]. A quality improvement project in Connecticut showed that collaboration between pediatric centers and community hospitals was able to significantly decrease CT scan use in children cared for in a community ED system [22].

Our data are also consistent with existing literature regarding the incidence of non-diagnostic studies in the community setting. Over 60% of our ultrasounds were non-diagnostic or did not visualize the appendix, yielding very minimal utility for these studies. These rates are similar to what other studies noted [5-7,23]. One study reported an over 70% rate of a nonvisualized appendix [6], with others reporting over 70% as non-diagnostic studies [5]. Various factors could contribute to this, including the quality of imaging obtained, the skill of the operator, and experience with performing this study on pediatric patients. In comparison to sonographers at academic institutions, those in community settings may have less exposure to pediatric ultrasound procedures, potentially resulting in difficulties in identifying or only partially visualizing the appendix. Additionally, certain patient-related factors can also contribute to inconclusive ultrasound readings, such as a higher body mass index, a retrocecal appendix position, normal appendix appearance, perforation, and inflammation at the distal tip [19]. Regardless, our data support the potential to improve upon ultrasound diagnostic capabilities, particularly in a community hospital setting where pediatrics may not be the primary patient population served.

This study was subject to various limitations. There are inherent limitations given the retrospective nature of the study, which we tried to mitigate by using standardized methods of data collection [24]. There are also many other variables that were not analyzed, such as the training of the radiologists interpreting the studies (pediatric radiology, general radiology) or the training and experience of sonographers performing the ultrasound studies. Finally, there is the selection bias of querying only cases of diagnosed acute appendicitis and not looking at all patients with imaging done to evaluate for appendicitis as the cause of abdominal pain, which may have resulted in ruling out appendicitis. These limitations are to be taken in the context of the study's strengths, which include its multi-center design, clearly defined objectives, defined outcomes, robust data collection practices, and clinical relevance to real-world practice.

## Conclusions

In this community hospital network, the majority of patients had multiple imaging modalities ordered to confirm the diagnosis of acute appendicitis. Less than half had an ultrasound ordered as the initial imaging modality, suggesting its use is not as widespread as academic data may indicate. Despite a positive diagnostic ultrasound, some patients went on to have a confirmatory CT scan, which provides further opportunity for improvement. We intend to use the findings of this study to help develop a standardized protocol for the workup of acute appendicitis throughout the hospital network.
